# Beyond the gender data gap: co-creating equitable digital patient twins

**DOI:** 10.3389/fdgth.2025.1584415

**Published:** 2025-04-30

**Authors:** Nora Weinberger, Daniela Hery, Dana Mahr, Stephan O. Adler, Jean Stadlbauer, Theresa D. Ahrens

**Affiliations:** ^1^ Institute for Technology Assessment and Systems Analysis (ITAS), Karlsruhe Institute of Technology (KIT), Karlsruhe, Germany; ^2^ Department of Digital Health Engineering, Fraunhofer Institute for Experimental Software Engineering IESE, Kaiserslautern, Germany

**Keywords:** digital patient twins, artificial intelligence, personalized medicine, gender data gap, ethical aspects, social implications, co-creation

## Abstract

Digital patient twins constitute a transformative innovation in personalized medicine, integrating patient-specific data into predictive models that leverage artificial intelligence (AI) to optimize diagnostics and treatments. However, existing digital patient twins often fail to incorporate gender-sensitive and socio-economic factors, reinforcing biases and diminishing their clinical effectiveness. This (gender) data gap, long recognized as a fundamental problem in digital health, translates into significant disparities in healthcare outcomes. This mini-review explores the interdisciplinary connections of technical foundations, medical relevance, as well as social and ethical challenges of digital patient twins, emphasizing the necessity of gender-sensitive design and co-creation approaches. We argue that without intersectional and inclusive frameworks, digital patient twins risk perpetuating existing inequalities rather than mitigating them. By addressing the interplay between gender, AI-driven decision-making and health equity, this mini-review highlights strategies for designing more inclusive and ethically responsible digital patient twins to further interdisciplinary approaches.

## Introduction

Digital patient twins (DPTs) are emerging as transformative tool in data-driven precision medicine, enabling highly individualized predictions. By leveraging artificial intelligence (AI) and real-time patient data, DPTs simulate disease progression, assess treatment responses, and refine clinical decision-making (1). Conceptualized as virtual models of patients, they integrate both population-based and patient-specific data and also could incorporate real-time inputs, e.g., from wearables (2, 3). Their potential lies in improving health monitoring and enabling personalized therapeutic strategies, while simultaneously optimizing treatment outcomes (4).

**Figure 1:**
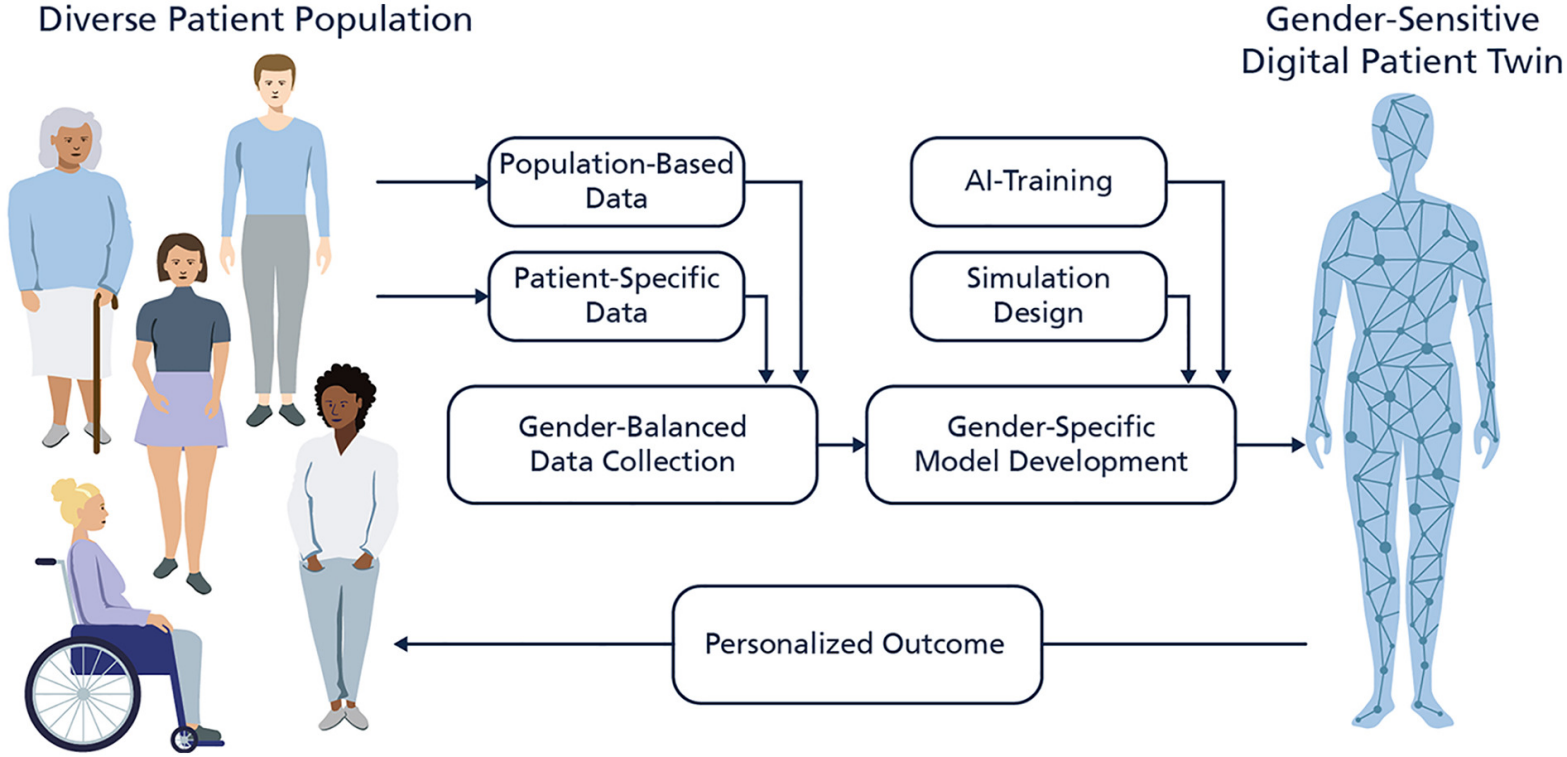
Illustration of the development of gender-sensitive digital patient twins (DPTs). The figure shows a gender-balanced data collection of population-based data and individual patient-specific data which is used to develop the model. The consideration of diverse patient populations during the development and validation phase enables a precise and personalized outcome by the underlaying artificial intelligence algorithm of the DPT.

However, most current DPT models have been developed with a critical oversight: *the lack of gender-sensitive design*. In this context, “gender-sensitivity” describes digital patient models explicitly designed to address this imbalance by incorporating sex- and gender-specific data, intersectional factors, and inclusive development processes. The dominant datasets used to train predictive models overrepresent male, white, and affluent populations, resulting in algorithmic biases that often fail to reflect the physiological and socio-medical diversity of patients (5). Since many DPTs rely on datasets derived from traditional clinical trials, which have historically underrepresented women and non-binary individuals, these biases particularly affect these groups, exacerbating existing health disparities and limiting the effectiveness of AI-driven healthcare solutions (6–9). This gender data gap—referring to the systemic underrepresentation of women and gender-diverse individuals in biomedical data and clinical studies,—continues to shape underlying digital infrastructures such as training data sets and artificial intelligence in ways that reduce model validity and exacerbate bias across clinical contexts. Figure 1 illustrates this process by showing how inclusive data collection and gender-sensitive model design can lead to more accurate and equitable DPTs. Beyond dataset biases, structural exclusions in clinical research and AI model development further contribute to these disparities. Many predictive models fail to capture key differences in drug metabolism, disease progression, and symptom manifestation, which can significantly impact treatment effectiveness (10–12). Without diverse training data and development teams, DPT-generated insights risk being inaccurate or even harmful for marginalized groups, reinforcing rather than mitigating disparities in healthcare.

Against this backdrop, this mini-review examines the technical foundation, medical relevance as well as ethical and social challenges of DPTs from an interdisciplinary perspective. This field is evolving rapidly, with conceptual, technical, and regulatory developments emerging across disciplines—from biomedical engineering to digital ethics and AI governance. While this analysis does not yet provide a systematic and exhaustive assessment, it aims to initiate a broader discussion on how gender-sensitive, intersectional, and co-creative approaches can shape the advancement of equitable healthcare solutions and personalized medicine.

## Data science perspective

The concept of digital twins originates in aerospace engineering in the 1960s, when NASA developed digital models of spacecraft to test various scenarios and monitor performance in real-time, improving mission efficiency and safety (13). This principle was later adapted to medicine, leading to the development of DPTs—data-driven models that replicate patient-specific biological and physiological processes to enhance for example diagnostics, treatment planning and disease progression analysis.

DPTs can be designed at various levels of abstraction and complexity, ranging from physiological function simulations, single-organs or to comprehensive patient models. Some implementations focus on specific pathological conditions, while others provide a broader representation of patient health allow early disease detection and risk prediction (14). In clinical applications, DPTs enable personalized treatment strategies by simulating responses to different therapeutic interventions, ultimately supporting precision medicine. The accuracy of DPTs relies on the integration of diverse and high-quality biomedical data sources, including vital signs, genetic profiles, metabolic markers, medical imaging, and patient history records. The ability to process and analyze multimodal data enhances predictive modeling and enables healthcare professionals to anticipate disease trajectories and optimize treatment plans. In radiology, for example, DPTs have demonstrated their value in improving diagnostic accuracy by correlating imaging data with patient-specific biomarkers (1). Similarly, in chronic disease management, they facilitate continuous remote monitoring, individualized interventions, as well as dynamic treatment adjustments, allowing healthcare professionals to preemptively address potential complications and reduce hospitalization rates (5).

However, while data integration enhances predictive power, DPTs face several technical challenges. The effectiveness of DPTs depends on data availability, quality, and standardization. Medical datasets are often fragmented across institutions, requiring advanced preprocessing techniques to harmonize diverse data inputs, which also drives (inter-)national harmonization projects. Moreover, computational models must be designed to accommodate inter-patient variability, ensuring robust predictions across different demographic and clinical profiles. One significant challenge in developing reliable DPTs is the lack of sufficient diversity in training datasets, particularly regarding sex- and gender-specific physiological variations. Drug dosages and side effects, for instance, can vary significantly between sexes due to differences in pharmacokinetics and pharmacodynamics (15). However, many existing DPT models are based on datasets that do not adequately capture these variations, limiting their ability to provide accurate, individualized treatment recommendations. Addressing these gaps requires more comprehensive data collection strategies that incorporate sex-specific and hormonal influences on disease progression and therapy responses. Beyond dataset limitations, the computational demands of DPTs continue to be a central challenge. The integration of machine learning and mechanistic modeling approaches requires high processing power and extensive validation methodologies to ensure reliability in clinical settings (16). Real-time simulation of complex biological processes remains particularly demanding, as nonlinear interactions between genetic, environmental and lifestyle factors must be incorporated into the models.

Future developments in AI-driven modeling, real-time data acquisition and computational scalability will be critical in enhancing the precision and adaptability of DPTs. Advancements in data integration frameworks and predictive analytics will play a key role in ensuring the effective deployment of DPTs across a wide range of medical applications.

## Medical perspective

DPTs have emerged as a transformative tool in personalized medicine, especially for managing chronic diseases with complex and long-term effects. A nationwide study from Denmark highlights the need for gender-based approaches as women are regularly later diagnosed than men and longitudinal disease trajectories for the same diagnosis differ between both sexes (17). By integrating patient-specific data, DPTs enable precision diagnostics, clinical decision support, individualized therapeutic strategies and potentially response prediction. One example for DPTs offering substantial benefits for gender medicine is cardiovascular disease, which remains the leading cause of mortality worldwide. However, sex-specific differences in symptom presentation contribute to delayed diagnoses, mismanagement, and higher mortality in women. Unlike men, who “typically” report chest pain as the primary symptom, women often experience “atypical” manifestations such as fatigue, nausea or back pain, resulting in delayed recognition and reduced urgency in seeking medical care (9). Indeed, women have a 2.2-fold higher 28-day mortality than men (18). DPTs could improve cardiovascular outcomes by integrating patient-specific physiological data and real-time monitoring. Wearable-integrated DPTs can track heart rate variability, blood pressure fluctuations and other biomarkers, providing personalized alerts for early warning signs (19). Additionally, in acute settings, DPT-assisted emergency diagnostics can support clinicians by identifying non-standard symptom patterns and prioritizing appropriate diagnostic pathways, reducing the risk of misdiagnosis (20). By refining predictive models for cardiovascular risk assessment, DPTs potentially contribute to earlier intervention and improved patient outcomes.

Another striking medical example of gender difference are immune diseases which affect women more frequently. In rheumatoid diseases different female-male ratios have been reported, ranging for instance from 9:1 for Sjögren's syndrome or 3:1 for rheumatoid arthritis (RA) (21). Women not only develop RA more frequently than men but also experience differences in disease progression and treatment response. However, standard diagnostic and therapeutic protocols often overlook these variations, leading to delayed diagnoses and suboptimal outcomes for female patients (22). DPTs mitigate these shortcomings by integrating sex-specific clinical data, improving diagnostic precision, and allowing for individualized treatment adaptations. Pharmacokinetic and pharmacodynamic properties differ significantly between sexes, influencing drug metabolism, efficacy, and safety (15). Leveraging resources such as the Janusmed Sex and Gender Database, DPTs can refine drug dosing strategies, minimize adverse reactions and enhance therapeutic outcomes (23, 24). This is particularly relevant for disease-modifying antirheumatic drugs like methotrexate, which have a higher risk of side effects in women (25). Rarely, men are more often affected than women as in the case of ankylosing spondylitis (21), showing the complexity of gender medicine perspectives.

In oncology, DPTs facilitate tailored treatment strategies by incorporating sex-specific biological variables that influence cancer progression and therapeutic efficacy. Tumor behavior varies between men and women due to hormonal influences, genetic predispositions and differences in immune response (26). Despite these differences, many clinical trials do not evaluate gender differences. In a meta-analysis on immune checkpoint inhibitors only 20 out of 7,133 studies reported sex-specific results (27), pointing towards higher efficacy in men. This highlights that women do not benefit in the same extent from innovative cancer therapies and that further protocol optimizations are needed. A novel study in mice shows that treatment timing with menstrual cycle impacts the efficacy significantly based on various effect from blood vessel diameters to molecular changes (28). Interestingly, a retrospective analysis of biobanked tissue and plasma samples from female breast cancer patients points to similar effects, opening a novel path for treatment individualization in women. By integrating all these potential factors, DPTs could support precision oncology approaches, optimizing treatment decisions and reducing gender-based disparities in cancer care. DPT-driven modeling allows for patient-specific adaptation of chemotherapy regimens, considering variability in drug metabolism and toxicity profiles. These insights help to minimize adverse effects and improve therapeutic response rates, ensuring more targeted and effective treatment strategies for different patient subgroups.

Overall, DPTs have potential to change medical care by moving beyond generalized clinical models to dynamic, patient-specific AI-driven simulations in various diseases, which goes well beyond the here described three examples. Their potential to systematically integrate complex interactions positions DPTs as bridging element in digital health solutions to close existing health disparities.

## Ethical and social science perspectives

The potential of gender-sensitive DPTs extends beyond technical advancements, offering a means to mitigate healthcare disparities (29–31). While traditional DPTs have improved diagnostic precision, they often fail to account for systemic biases as they rely on homogeneous datasets primarily reflecting male, urban, and middle-class populations (32). This lack of diversity has led to biased predictions, disadvantaging groups whose health outcomes are shaped by gender, race or socio-economic factors (15, 33). Gender-sensitive DPTs seek to correct these distortions by integrating intersectional perspectives and more representative data, enhancing diagnostic accuracy and fostering trust among historically underserved populations. One of the key ethical challenges of traditional DPTs has been the opacity of predictive algorithms, where patients and clinicians lack insights into how decisions are generated. Gender-sensitive models, by contrast, aim to increase transparency, enabling greater interpretability of predictions and ensuring that AI-driven recommendations can be contextualized within individual medical histories (7). This shift not only fosters greater trust in digital tools but also strengthens clinician-patient relationships by maintaining medical oversight rather than shifting authority toward algorithmic decision-making.

Despite these advancements, new ethical risks emerge by gender-sensitive DPTs, particularly regarding privacy and data security. Gender-sensitive DPTs require highly granular health data, including hormonal fluctuations, metabolic markers, and socio-economic factors, which increases the risk of data breaches and unauthorized access (26). Without strict regulatory frameworks, such detailed datasets could be misused or exacerbate existing biases in healthcare access. A further challenge lies in ensuring informed consent in AI-driven decision-making. Patients and clinicians must not only understand how DPTs generate predictions but also recognize their limitations. Notably, the human-machine interaction is quite complex, and the combination of AI and humans does not always guarantee better results, especially for decision making (34). If predictive analytics are treated as neutral and infallible decision-making tools, patients may feel pressured to follow AI-generated recommendations without questioning them. This can undermine patient autonomy and reinforce a form of “algorithmic paternalism,” where medical decisions appear to be dictated by algorithms rather than informed, shared decision-making between patients and clinicians [cf (35)., for example]. This ethical dilemma becomes particularly tangible in the field of cardiovascular diagnostics: when DPTs trained on male-dominated datasets are deployed without gender-sensitive adjustments, they may fail to identify “atypical” symptom patterns in women—such as fatigue or abdominal pain. If physicians rely uncritically on such biased predictions, this could lead to missed diagnoses and undermine patient safety. Ethically, this raises urgent concerns about how automated decisions interact with implicit gender norms in clinical judgment and how accountability is distributed between humans and machines.

Ensuring that DPTs support rather than dictate medical decisions is crucial to maintaining autonomy in clinical practice. Even within gender-sensitive models, biases can persist if historical inequities are embedded in training data. For instance, cardiovascular risk assessments remain disproportionately based on male-centered data, contributing to underdiagnosis of heart disease in women (15). Continuous bias detection and adaptive learning mechanisms are therefore essential to prevent DPTs from perpetuating disparities instead of mitigating them. Beyond algorithmic concerns, broader structural inequalities such as the digital divide pose further ethical risks. While DPTs offer potential for personalized medicine, they require technological access which may not be available to all patients. Socio-economically disadvantaged groups often face barriers to digital health tools, limiting the equitable distribution of these innovations (36). Ensuring that DPTs are designed for accessibility, with low technological barriers and equitable integration strategies, is essential to prevent new forms of healthcare exclusion.

## Participatory and intersectional perspectives

As described above, the development of DPTs opens new possibilities for personalized medicine, yet their potential can only be fully realized if they are designed to be inclusive and reflective of diverse populations. Co-creation methodologies play a crucial role in ensuring that these technologies are adapted to the needs of different user groups, whether patients, healthcare providers or researchers. Rather than being shaped solely by developers and clinicians, DPTs must integrate the perspectives of all relevant stakeholders from the outset (37, 38). This participatory approach makes it possible to align technological development with the realities of medical practice and patient care, ensuring that the models are not only accurate but also usable in different healthcare contexts.

A key aspect of inclusive DPT development is the integration of intersectional perspectives. Healthcare outcomes are shaped by a complex interplay of biological, social, and economic factors, meaning that a one-size-fits-all approach to predictive modeling risks overlooking critical differences in disease manifestation and treatment response (22, 25). Traditional medical models have frequently been based on data from predominantly male and homogenous populations, which can lead to biased diagnostic tools and misrepresentation of conditions that affect marginalized communities. Co-creation processes allow for the identification and active mitigation of such biases by involving those most affected, such as patients and healthcare professionals from marginalized groups, directly in the development and design of the technology. Beyond improving the representativeness of data, co-creation fosters a more needs-oriented approach to DPT development. Depending on the context, DPTs may serve different functions, from predictive diagnostics, personalized treatment planning to clinical decision support as well as the support of research. Their usability is therefore contingent for ensuring that they not only reflect the medical realities of diverse populations, but also the requirements of all stakeholders that interact—whether as direct users, decision-makers or healthcare professionals. Involving stakeholders in the development process enhances acceptance and usability, as it allows for an early and ongoing adaptation of the DPT models to specific medical, regulatory and practical constraints (37, 38). Participatory approaches thus offer multiple benefits: they improve clinical applicability by aligning DPT functions with real-world workflows; they enhance trust and acceptance among users; and they reduce the risk of design flaws or blind spots that might otherwise remain unaddressed. In this sense, co-creation is not only a normative ideal but a practical prerequisite for building equitable and effective digital health tools.

Another major challenge in achieving equitable access to DPTs is the digital divide, which disproportionately affects individuals from lower-income backgrounds and elderly populations. Limited access to digital health technologies and lower levels of digital literacy can significantly impact the ability of these groups to benefit from innovations such as DPTs. If these disparities are not addressed, there is a risk that digital healthcare advancements will further marginalize those already facing barriers to medical care (36). To ensure that DPTs serve all populations equitably, their design must prioritize accessibility, including user-friendly interfaces, multilingual options, and integration with non-digital healthcare systems. Additionally, embedding social determinants of health into DPT development could improve their predictive accuracy, allowing these tools to account for disparities rather than inadvertently reinforcing them.

## Conclusion

DPTs represent a significant advancement in precision medicine, offering new opportunities for personalized diagnostics and therapy. Although they have been piloted in a variety of clinical contexts, their practical implementation in clinical workflows remains currently limited and often exploratory. Their development still faces fundamental challenges, particularly regarding gender bias and health equity. This review outlines key challenges and interdisciplinary perspectives, serving as an initial step toward more comprehensive and systematic investigations.

Addressing such challenges requires an interdisciplinary approach that integrates insights from software engineering, medicine, data science, social sciences, and ethics. The technical perspective emphasizes the need for algorithmic transparency, dataset diversity, data quality, as well as bias mitigation to improve reliability of DPTs. From a medical perspective, gender-sensitive models are critical for enhancing diagnostic accuracy, tailoring treatment plans, and improving pharmacological safety, particularly in fields such as cardiology, oncology, or other chronic diseases. Ethical and social science perspectives highlight the risks of algorithmic bias, privacy violations, and the reinforcement of digital divides, especially when AI-driven healthcare systems lack transparency and fail to prioritize equitable access.

However, interdisciplinary collaboration alone is insufficient. While it enhances technical robustness and medical applicability, it does not ensure that DPTs meet the real-world needs of diverse patient populations and healthcare professionals. To fully integrate a gender-sensitive and equitable design, a transdisciplinary, participatory approach is essential. Co-creation is not merely an instrument for collecting feedback—it is a necessary condition for developing DPTs that accurately reflect the lived experiences of patients, clinicians, and other stakeholders. Direct engagement with those affected by AI-driven decision-making is crucial for five key aspects:

• Improving the real-world relevance of DPTs: Ensures that predictive models align with actual clinical workflows and patient needs.
• Enhancing trust and acceptance: Enables patients and clinicians to understand, use, and trust AI-assisted diagnostics and treatment recommendations.
• Eliminating “user blind spots” in technical development: Prevents misaligned assumptions between algorithmic design and healthcare practices.
• Empowering patients: Gives them greater control over their digital representations, rather than treating them as passive data sources.
• Preventing unintended biases: Ensures that new discriminatory patterns do not emerge through AI-based decision-making.

To operationalize a gender-sensitive DPT framework, a structured, participatory workflow is needed that combines technical precision, medical applicability, and social inclusivity (see Table 1).

**Table 1:** Actions for gender-sensitive digital patient twin (DPT) implementation.

Workflow stage	Key considerations for implementation
1. Data collection	Integrate diverse, intersectional datasets, capturing sex, gender, ethnicity, age, and socioeconomic factors to reduce bias and improve inclusivity.
2. Ethical framework design	Establish privacy, security, and informed consent protocols, with feedback mechanisms to ensure transparency and patient autonomy.
3. Model development	Collaborate with interdisciplinary and diverse teams and affected stakeholders to design adaptive algorithms that incorporate gender-specific health markers and intersectional health variables.
4. Co-creation & participatory design	Actively engage patients, healthcare providers, and community representatives in all development cycles to ensure DPTs align with real-world needs and patient experiences.
5. Bias testing and mitigation	Implement regular bias audits, ensuring that gender- and intersectional fairness metrics are met across diverse patient populations.
6. Clinical implementation	Train clinicians in (gender-sensitive) AI usage, ensuring that DPT-assisted decision-making accounts for diverse health profiles.
7. Continuous evaluation	Establish long-term monitoring strategies, integrating user feedback, clinical validation, and regulatory oversight to refine models and detect emerging biases.

This structured framework outlines concrete actions for integrating gender sensitivity into the development and deployment of DPTs. However, ensuring that these measures are effectively implemented and sustained over time requires more than technical adjustments—it necessitates a fundamental rethinking of how digital health technologies are designed, governed, and integrated into medical practice.

Simply embedding more diverse datasets or refining AI models does not automatically result in equitable outcomes. Without a participatory and transdisciplinary approach, gender-sensitive algorithms risk being developed in isolation from the very people they are meant to serve. Thus, the success of these measures depends on embedding co-creation not just as a development step, but as an ongoing governance principle.

The future of DPTs lies not only in more sophisticated AI but in more inclusive, transparent, and patient-centered design processes. Achieving this vision requires:

• Regulatory commitments that enforce gender-sensitive and intersectional data standards.
• Stronger interdisciplinary and transdisciplinary collaborations, bringing together e.g., technical experts, medical professionals, ethicists, and affected communities.
• Participatory governance models that ensure continuous stakeholder involvement, bias monitoring, and adaptive learning.

International policy frameworks are beginning to reflect these priorities. The European Union's AI Act, for example, classifies AI applications in healthcare as high-risk technologies, requiring safeguards against algorithmic bias and promoting transparency. Likewise, the WHO's ethical guidance on AI for health calls for inclusive, gender-sensitive, and context-aware design standards. Such policy instruments provide a crucial orientation for aligning DPT development with the principles of equitable precision medicine. Without such measures, DPTs risk reinforcing rather than reducing existing health inequities. However, with inclusive, evidence-based, and participatory development, these technologies can serve as a powerful tool for equitable, personalized medicine.
